# Slipped Capital Femoral Epiphysis as a Presentation of Underlying Metabolic Disorders: Pseudohypoparathyroidism and Juvenile Hypothyroidism

**DOI:** 10.7759/cureus.13775

**Published:** 2021-03-09

**Authors:** Rainel Zelaya, Anthony Zarka, Douglas Byerly

**Affiliations:** 1 Radiology, Brooke Army Medical Center, Fort Sam Houston, USA; 2 Pediatric Radiology, Baylor College of Medicine/Children’s Hospital of San Antonio, San Antonio, USA; 3 Department of Radiology, Wilford Hall Ambulatory Medical Center, San Antonio, USA

**Keywords:** scfe, pseudohypoparathyroidism, hypothyroidism, klein, salter-harris, greulich, pyle

## Abstract

Slipped capital femoral epiphysis (SCFE) is an abnormality of the proximal femoral physis typically occurring in adolescents and most commonly associated with obesity, although its exact etiology is unknown. In addition to obesity, other associations and predisposing factors proposed in the literature include trauma, vascular injury or compromise, and immunologic, genetic, and metabolic conditions. While not common, metabolic conditions are known to predispose to SCFE and it is essential for radiologists to recognize SCFE as a possible initial presentation of an underlying metabolic disorder. Understanding imaging findings and identification of atypical presentations of SCFE by radiologists can assist clinicians in guiding workup and lead to expedited treatment to prevent worsening outcomes associated with developmental delay.

## Introduction

Slipped capital femoral epiphysis (SCFE) is a Salter-Harris type 1 injury involving the proximal femoral physis which results in eventual slippage of the proximal femoral epiphyses in relation to the metaphysis. The exact etiology of SCFE is disputable. However, both biomechanical and biochemical changes that occur at puberty are generally accepted etiologies. Age of presentation is affected by gender, with males typically presenting between the ages of 10-17 and females presenting slightly earlier between the ages of 8 and 15 [1]. Metabolic imbalances related to the thyroid, parathyroid, and pituitary glands such as hypothyroidism, pseudohypoparathyroidism, and hyperparathyroidism are known to be associated with SCFE [2]. In this case report, we present two patients presenting with imaging findings consistent with SCFE at the extremes of age than are typically seen with this condition, attributed to hypothyroidism and pseudohypoparathyroidism.

## Case presentation

Case 1

**Figure 1:**
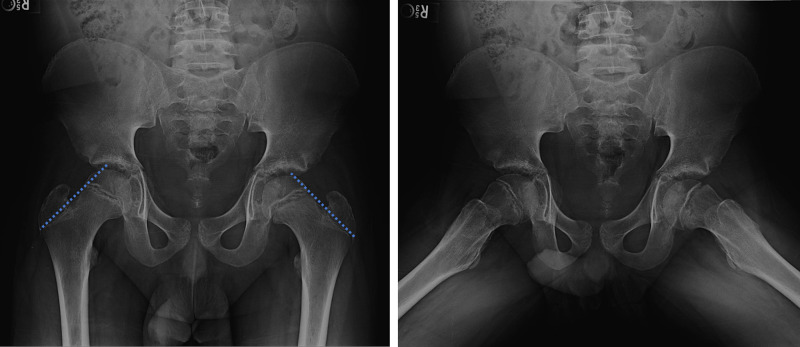
Plain radiographic AP (left image) and frog leg (right image) views of the bilateral hips. Suspected left SCFE in a 19-year-old male presenting with hip pain. AP view demonstrates lines of Klein (blue dotted lines). The left line does not intersect with the femoral epiphysis and passes above the left femoral head, supporting the diagnosis. There is slight intersection of the right femoral head by the line of Klein. Frog leg views demonstrate the posteromedial translation of the left proximal femoral epiphysis in relation to the metaphysis. The left femoral physis appears abnormally widened and irregular on both images. Note that the visualized osseous structures demonstrate abnormal delayed skeletal maturation, with no significant closure of the femoral physes or apophyses, inconsistent with reported age. AP, anteroposterior; SCFE, slipped capital femoral epiphysis

A 19-year-old male basic military trainee reported chronic, intermittent bilateral hip and groin pain since childhood, which acutely worsened over the past two weeks related to increased physical training. He reported mild discomfort associated with long periods of walking which increased with running, left greater than right. Pain decreased but persisted at rest. The patient’s pain resulted in walking with a limp. Physical examination was notable for joint pain in both hips with active and passive motion, which worsened with weightbearing. The patient appeared younger than his stated age. Bilateral hip radiographs were obtained with frog leg views (Figure 1). Findings were concerning for left SCFE, possible right SCFE, and abnormal delayed skeletal maturation without significant closure of the femoral physes or apophyses.

**Figure 2:**
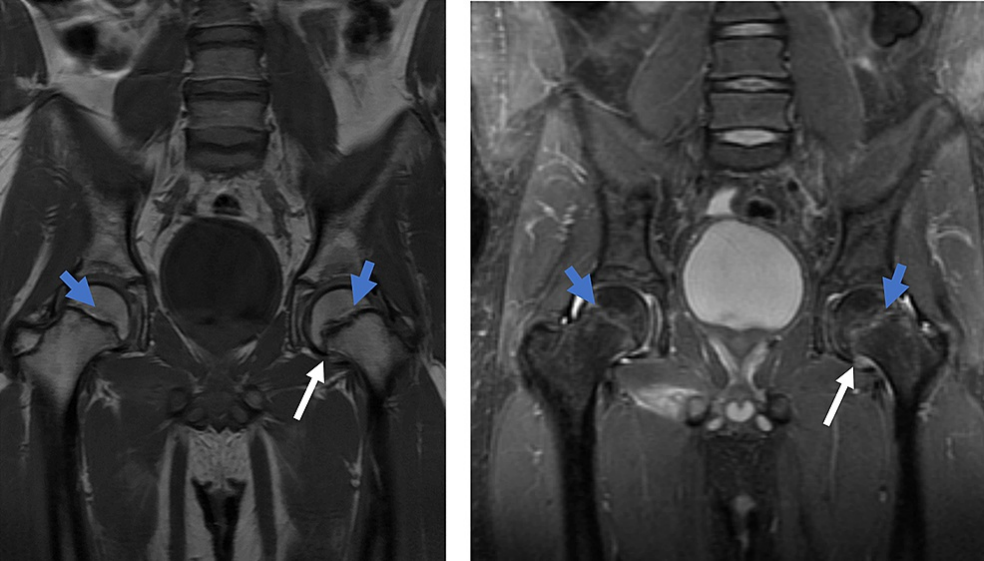
MRI of the bilateral hips. Coronal T1 (left image) and coronal STIR (right image) images of the bilateral hips demonstrate left greater than right periphyseal edema (blue arrows) characterized by mild STIR hyperintense and mild T1 hypointense signal with slip of the left femoral epiphysis (white arrows). Although there is some periphyseal edema of the right femur, epiphyseal slippage is not evident. Open physes are again seen, representing delayed closure in this 19-year-old patient. MRI, magnetic resonance imaging; STIR, short TI inversion recovery

The radiologist recommended orthopedic referral and obtaining a bone age assessment to estimate the amount of delay in skeletal maturation. He was subsequently admitted by the orthopedic department for treatment with bilateral surgical pinning. Magnetic resonance imaging (MRI) of the bilateral hips was ordered prior to surgery, which further supported findings consistent with left SCFE (Figure 2).

**Figure 3:**
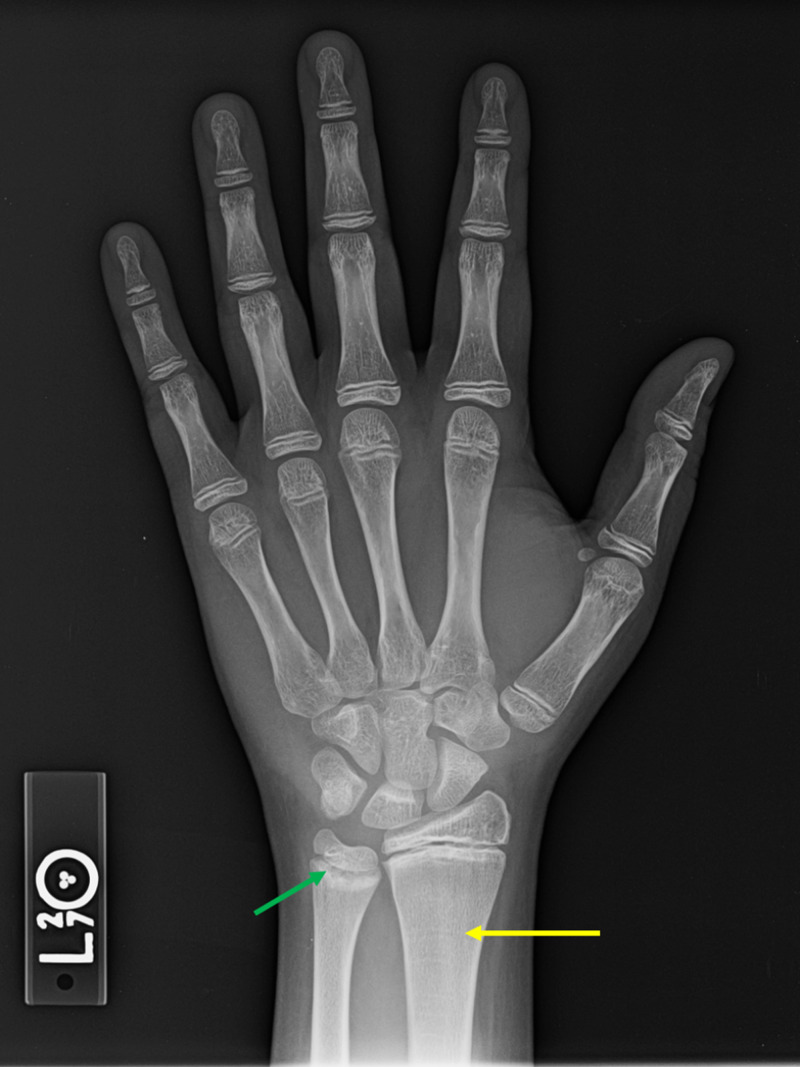
Bone age radiographs of the left hand and wrist. The patient most closely resembles the Greulich and Pyle male standard for 13 years and 6 months (162 months). The patient’s chronologic age during time of this study was 19 years and 2 months (230 months). Note the multiple growth recovery lines within the distal radial metadiaphysis (yellow arrow). There is relative increased density and mild cupping of the distal ulnar metaphysis (green arrow).

Subsequent clinical follow-up initiated by his primary provider and the referred endocrinologist were notable for an elevated thyroid-stimulating hormone above 160 mcIU/mL, a low level of free thyroxine, and an elevated serum thyroperoxidase antibody level of 765 IU/mL, findings consistent with hypothyroidism likely secondary to Hashimoto thyroiditis. Further history obtained from the endocrinologist revealed symptoms consistent with hypothyroidism: fatigue, constipation, increased sleep, and problems with losing weight but without cold intolerance. A bone age study demonstrated a markedly delayed bone age, consistent with Greulich and Pyle male standard for 13 years and 6 months, incongruent with the patient’s chronologic age of 19 years and 2 months (Figure 3).

Following surgical pinning the patient was started on oral levothyroxine which was subsequently titrated as needed and demonstrated marked clinical improvement of his previous symptoms associated with hypothyroidism and with resolution of hip pain. Initial follow-up bone age studies demonstrated mild progression of physeal closure (not shown).

Case 2

**Figure 4:**
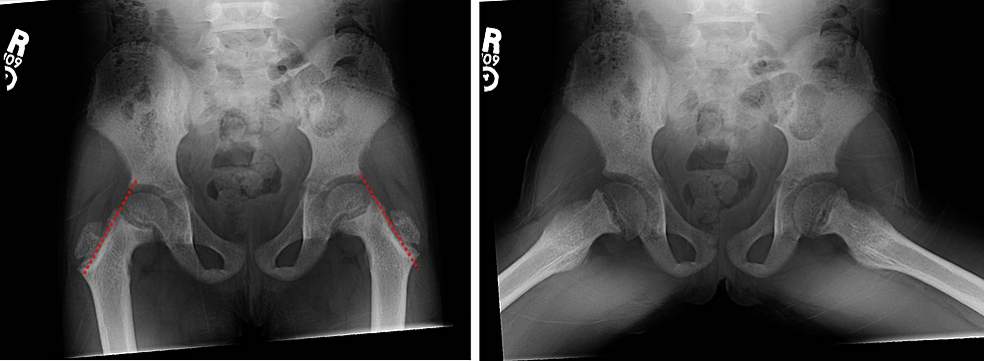
Plain radiographic AP (left image) and frog leg (right image) views of the bilateral hips. Six-year-old female presenting with limping. AP view demonstrates lines of Klein (red dotted lines) positioned above the femoral heads without intersection of the epiphysis. Frog leg views demonstrate the posteromedial translation of the proximal femoral epiphysis in relation to the metaphysis. Note the abnormal bone with coarsened trabecula, osteopenia, irregular widening of all the growth plates, and irregularity to the peripheral margins of the iliac crests. These findings were concerning for an underlying metabolic bone disease or endocrinopathy. AP, anteroposterior

A six-year-old female presented to her pediatrician with her father due to limping for two weeks. The child denied hip pain at the clinic visit but reported hip pain when sitting on the floor with her legs crossed for long periods of time. Physical examination of the hips was unremarkable, except for the inability to duck walk, and losing balance frequently with no pain report. Bilateral hip radiographs were obtained with frog leg views, demonstrating findings consistent with right SCFE, with possible left SCFE (Figure 4). Additionally noted were osseous findings consistent with an underlying metabolic or endocrine disease.

**Figure 5:**
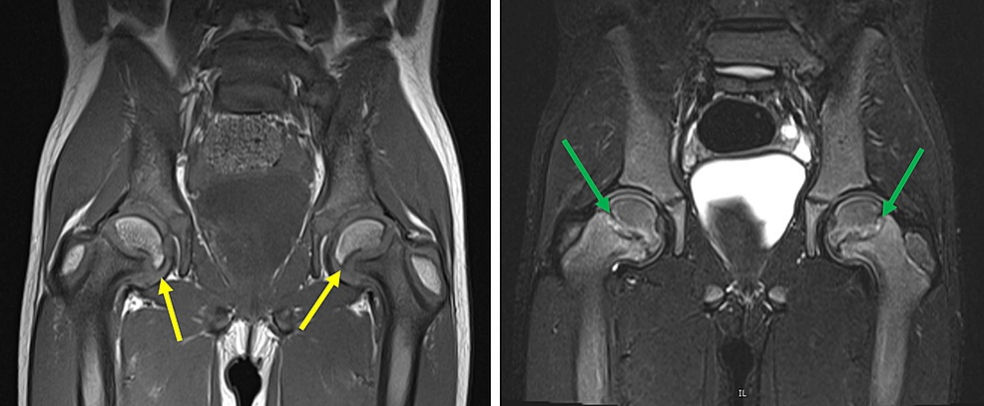
MRI of the bilateral hips. Coronal T1 (left image) and coronal STIR (right image) images of the bilateral hips redemonstrate bilateral right greater than left SCFE (yellow arrows). Accompanying moderate edema is noted along the metaphyseal and epiphyseal margins of the proximal femoral physes. Note bilateral coxa vara and irregular appearance of both proximal femoral metaphyses with mild widening and irregularity of the physes (green arrows), concerning for an underlying metabolic bone disease. MRI, magnetic resonance imaging; STIR, short TI inversion recovery; SCFE, slipped capital femoral epiphysis

The patient was referred to orthopedics and placed on non-weightbearing status, with eventual workup for bilateral surgical pinning. An MRI of the bilateral hips obtained prior to surgery demonstrated mild bilateral, right greater than left, SCFE with redemonstrated findings supporting metabolic disease (Figure 5).

She was additionally referred to a pediatric endocrinologist after lab findings concerning for pseudohypoparathyroidism, with an elevated parathyroid hormone (PTH) at 607 pg/mL, a mildly decreased serum calcium at 8.6 mg/dL, and an elevated serum phosphate at 6.2 mg/dL. Additional testing revealed metabolic PTH resistance. The clinical presentation was consistent with a de novo case of pseudohypoparathyroidism type 1b, which is characterized principally by PTH resistance. Calcium/calcitriol treatment was started, and the family was referred for genetic counseling. The patient had a normal postoperative course in the following months but was lost to follow-up afterward.

## Discussion

Pediatric patients with hip pain or limping with subsequent radiographs should be evaluated for SCFE, and the remainder of the visualized skeletal structures must also be evaluated for additional abnormalities, as were present in both cases of patients presenting outside the typical age range of SCFE. Additional evaluation of remaining bone structures in addition to recognizing SCFE led to the workup of underlying endocrine disorders (hypothyroidism and pseudohypoparathyroidism type 1b), which may both predispose patients to develop SCFE [3-5].

**Figure 6:**
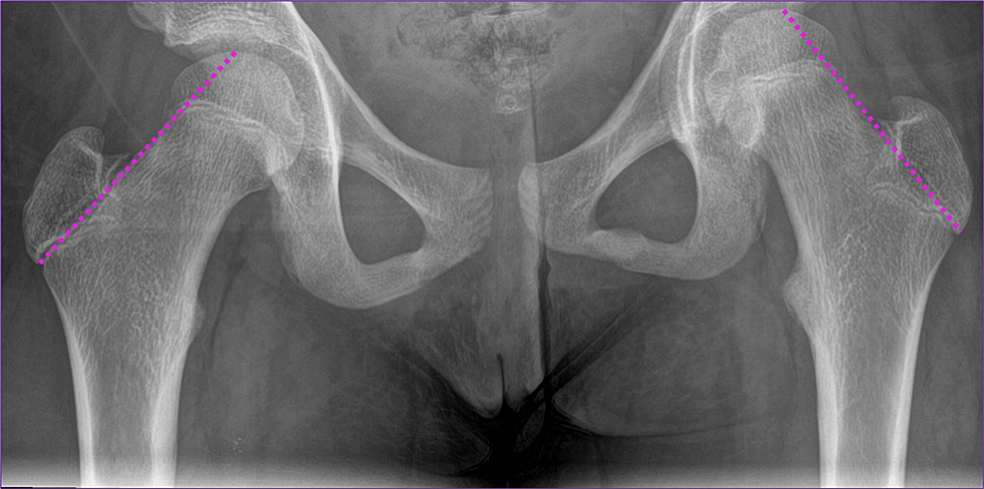
Normal AP hip radiograph of a 12-year-old female. Demonstrated are normal lines of Klein (pink dotted lines) drawn appropriately about the lateral proximal femurs. Note how both Klein lines intersect the femoral epiphyses. AP, anteroposterior

Radiographic evaluation of pediatric patients with hip pain should include lines of Klein, a line along the superior border of the femoral neck which should cross the lateral part of the superior femoral epiphysis on an anteroposterior radiograph [6] (Figure 6). SCFE is diagnosed if the line does not cross the epiphysis and passes above the femoral head, as was present in both cases [7]. Other radiographic findings supporting the diagnosis depend on the presentation. Pre-slip images may demonstrate a widened physis along with irregularity and haziness of the physeal margins, and subsequently, a posteromedial acute slip which may be better visualized on the frog leg view [1].

Radiographs are the primary imaging modality for assessment of pediatric hip pain and SCFE, and recognition of expected findings of SCFE is essential for radiologists. Ultrasound findings are not specific and may demonstrate a femoroacetabular joint effusion, and possibly show malalignment between the epiphysis and metaphysis of the proximal femur. Although CT demonstrates the typical epiphyseal/metaphyseal malalignment, ionizing radiation limits its use in pediatric patients and is therefore not recommended. MRI is helpful in the acute stage, typically demonstrating the malalignment in addition to increased T2 signal and corresponding low T1 signal, representing marrow edema surrounding the epiphyseal border [8]. MRI findings may also include an associated hip joint effusion as well as document preservation of blood flow to the femoral epiphysis if contrast is administered.

As both cases demonstrated, metabolic disturbances may predispose to SCFE, and examining osseous structures in patients with SCFE for signs of metabolic abnormalities or inconsistencies with patient age may be the first step in directing the appropriate workup to reverse the underlying cause. Several findings may suggest metabolic disorders, and pseudohypoparathyroidism shares many of the same skeletal features seen with other disorders such as Rickets, hyperparathyroidism, and osteomalacia [9]. These include metaphyseal irregularities, widening of the growth plates, osteopenia with decreased cortical and trabecular bone, coxa vara, and SCFE, most of which were seen in our second case.

Skeletal findings associated with congenital hypothyroidism are well documented in the literature; however, skeletal findings of juvenile hypothyroidism are less defined due to its scarcity. The most common cause of hypothyroidism is Hashimoto thyroiditis, as seen in our first case. Documented findings in the literature have included failed epiphyseal closure consistent with delayed bone age, dwarfism, and thickened bands at the metaphyseal ends, also known as growth recovery lines [10,11]. Other than dwarfism, the remainder of these findings were noted in our first case. If delayed bone age is suspected, dedicated bone age studies may be used to confirm the diagnosis [12,13].

Treatment of SCFE typically involves surgical pinning without reduction of the malalignment as this could compromise blood flow to the femoral epiphysis [14,15]. Lack of treatment may result in significant morbidity, including complications such as osteoarthrosis, avascular necrosis, chondrolysis, and limb length discrepancy [16,17]. Correction of the underlying metabolic disturbances is generally expediently started upon discovery to prevent further complications related to developmental delay [18,19].

## Conclusions

Diagnosis of SCFE is crucial in the imaging evaluation of pediatric patients with hip pain or limping and may prevent significant morbidity directly related to SCFE, while also potentially unmasking an underlying metabolic disorder such as hypothyroidism or pseudohypoparathyroidism, as demonstrated by our two case presentations of patients presenting outside the age range that is typically seen with SCFE. It is also equally important for radiologists to carefully inspect the remaining visualized skeletal structures and consider bone age studies to substantiate the diagnosis of a metabolic derangement.
